# Study of the Bus Dynamic Coscheduling Optimization Method under Urban Rail Transit Line Emergency

**DOI:** 10.1155/2014/174369

**Published:** 2014-11-04

**Authors:** Yun Wang, Xuedong Yan, Yu Zhou, Jiaxi Wang, Shasha Chen

**Affiliations:** State Key Laboratory of Rail Traffic Control and Safety, School of Traffic and Transportation, Beijing Jiaotong University, Beijing 100044, China

## Abstract

As one of the most important urban commuter transportation modes, urban rail transit (URT) has been acting as a key solution for supporting mobility needs in high-density urban areas. However, in recent years, high frequency of unexpected events has caused serious service disruptions in URT system, greatly harming passenger safety and resulting in severe traffic delays. Therefore, there is an urgent need to study emergency evacuation problem in URT. In this paper, a method of bus dynamic coscheduling is proposed and two models are built based on different evacuation destinations including URT stations and surrounding bus parking spots. A dynamic coscheduling scheme for buses can be obtained by the models. In the model solution process, a new concept—the equivalent parking spot—is proposed to transform the nonlinear model into an integer linear programming (ILP) problem. A case study is conducted to verify the feasibility of models. Also, sensitivity analysis of two vital factors is carried out to analyze their effects on the total evacuation time. The results reveal that the designed capacity of buses has a negative influence on the total evacuation time, while an increase in the number of passengers has a positive effect. Finally, some significant optimizing strategies are proposed.

## 1. Introduction

In the past few decades, urban rail transit (URT) has become one of the most important urban commuter transportation modes [1]. Due to the advantages of large capacity, fast speed, and high punctuality, more and more commuters are inclined to choose URT as their trip mode [2]. However, the frequency of accidents on URT systems has increased greatly, harming passenger safety and causing severe traffic delays. Moreover, the consequences of accidents in URT are often much more serious than those occurring elsewhere. The reason for this is, firstly, that URT involves dense passenger flow, such as Beijing, the traffic volume of which at peak hours can be as high as ten million [3]. Secondly, most of the URT systems are in underground spaces with closed environments [4]. When accidents happen, the available passageways to safety for passengers can be extremely narrow, which may result in disasters.

With the continued increase in accidents, security operation of URT system has been a hot research issue among operation departments and scholars. Once accidents happen, the most important task is to evacuate passengers to a safe space. However, the lack of contingency plans can make this very difficult. The previous literature has pointed out that contingency plans and safety evaluations should be carried out throughout the process of planning, designing, constructing, and operating URT [5–7].

Many personnel evacuation models have been developed to quantify the evacuation capacity or velocity and applied to the design of URT passenger evacuation. Previous studies related to passenger evacuation in URT emergencies mainly involve the following four aspects: fire emergency, equipment failure, trampling accidents, and unexpectedly large passenger flow [8]. For fire emergencies, Li et al. [9] and Yang et al. [10] carried out a computer simulation of the personnel evacuation progress based on the occupant evacuation dynamic model. The results showed that only when RSET (the real evacuation time from the start of the fire to the end of the evacuation) was less than ASET (the standard evacuation time, usually more than six minutes in the case of fire) could passengers be evacuated safely. Regarding equipment failure, Cheng and Yang [11] established an Emergency Evacuation Capacity (EEC) model to estimate the evacuation capacity of a subway station by analyzing key influential factors. Fridolf et al. [12] performed a train evacuation experiment to study the effects of different train exit configurations on the flow rate of people passing through an exit inside a tunnel. The results revealed that the height of the door, the material of the tunnel floor, the presence of emergency ladders, lighting, and the population density outside the train all significantly affected the flow rate. For trampling accidents, Liu et al. [13] used the Data Envelopment Analysis (DEA) method to assess the risk of trampling accidents in subway stations. However, most of this research has focused on the capacity or velocity of the evacuation of passengers from URT stations, while specific ways of evacuating passengers from the stations and achieving transport continuation have been neglected. In fact, only a few studies have considered the cooperation between the rail transit system and the ground transportation in an emergency situation [14, 15].

In this paper, a new method, dynamic coscheduling of buses, is proposed for evacuating passengers from dangerous places to safe areas more efficiently. Moreover, in the model solution process, a new concept of the equivalent parking spot is presented to transform the nonlinear problem into an integer linear programming (ILP) problem [16]. Because of the considerable uncertainty about the actual values of the model parameters, sensitivity analysis of model performance is necessary, especially for nonlinear models [17]. Saltelli and Annoni defined sensitivity analysis as the study of changes in the information flowing into or out of the model [18]. Sensitivity analysis is considered as good modeling practice when performed as part of model verification and has been widely used to assess quantitative models in many studies [19].

This paper is organized as follows. In Section 2, the definition of the dynamic coscheduling of buses in the case of an URT line emergency is introduced.  Section 3 describes the model development process, which includes three parts: the model assumptions, building, and solution. In Section 4, a case is presented and sensitivity analysis of two vital factors is carried out. Finally, Section 5 provides conclusions.

## 2. Definition of Dynamic Coscheduling of Buses

When an emergency occurs in an URT system, influencing the whole URT line, the service will be unavailable or the transport capacity will be insufficient. Therefore, once an emergency happens, a dynamic coscheduling scheme for buses should be formulated based on the real-time situation of passenger flow volume and reserved number of buses in each parking spot. The dynamic coscheduling scheme for buses is used to evacuate stranded passengers from subway stations, ensuring the safety of the passengers and eliminating passenger delay. The evacuation of passengers is achieved by dispatching buses from bus parking spots to rail transit stations.

In this paper, there are two kinds of evacuation destination, namely, rail transit stations and surrounding bus parking spots. The dynamic coscheduling problem for buses can thus be defined in the two cases, as follows.

### 2.1. When the Evacuation Destinations Are the Rail Transit Stations

**Figure 1:**
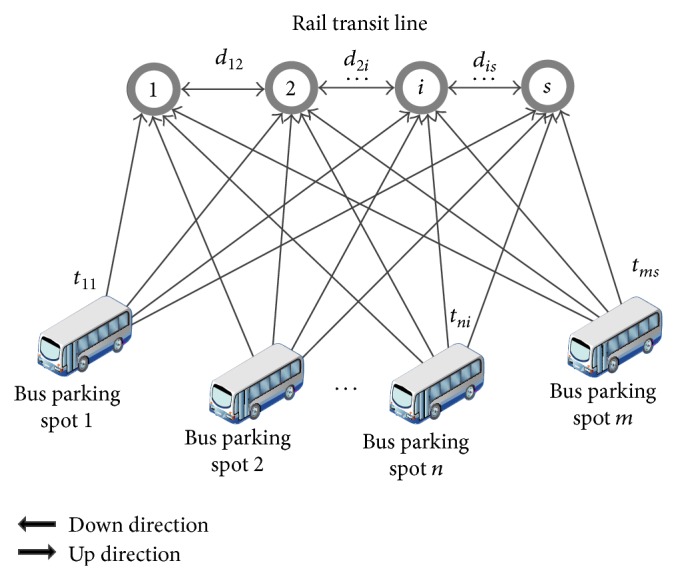
Topological structure when destinations are the rail transit stations.

When the evacuation destinations are the rail transit stations, the task of the dispatched buses is to evacuate passengers stranded at one rail transit station to their original destination.  Figure 1 shows the topological structure of this problem. As is shown, the direction from Station 1 to Station *n* is defined as the up direction, and the opposite direction is the down direction. The number of bus parking spots is denoted by *m* while the number of available buses in bus parking spot *n* is denoted by *N*_*n*_.

In the up direction, parking spot *n* dispatches buses to rail transit station *i*. After the passengers get on, the buses run to station *n* from station *i* and passengers can get off along the way. When the last passenger gets off at station *n*, the bus returns to its original bus parking spot and completes the evacuation task in the up direction. Similarly, in the down direction, parking spot *n* again dispatches buses to rail transit station *i*. Unlike in the up direction, however, after the passengers get on, the buses run to station 1 from station *i* and passengers again get off along the way. When the last passenger gets off at station 1, the bus returns to its parking spot and completes the evacuation task in the down direction.

The advantage of this evacuation method is that passengers can arrive at their original destination without changing their travel route or transferring to other traffic modes. This method is suitable for those rail transit lines with short distances between stations and low operating mileages.

### 2.2. When the Evacuation Destinations Are Bus Parking Spots

In the URT system, there is a kind of rail transit line that is mainly used to connect the central urban area to surrounding satellite towns and guide urban development, such as the Beijing Subway's YIZHUANG Line and BATONG Line. These kinds of lines are always constructed in towns and industrial parks where it is inconvenient for passengers to travel and the road infrastructure is inadequate. When an emergency occurs on this kind of rail transit line, due to the long distances between stations, it would take a long time for the dispatched buses to complete the evacuation from the originating station to the terminal station. Therefore, there is a huge advantage in choosing bus parking stops as the evacuation destinations.

**Figure 2:**
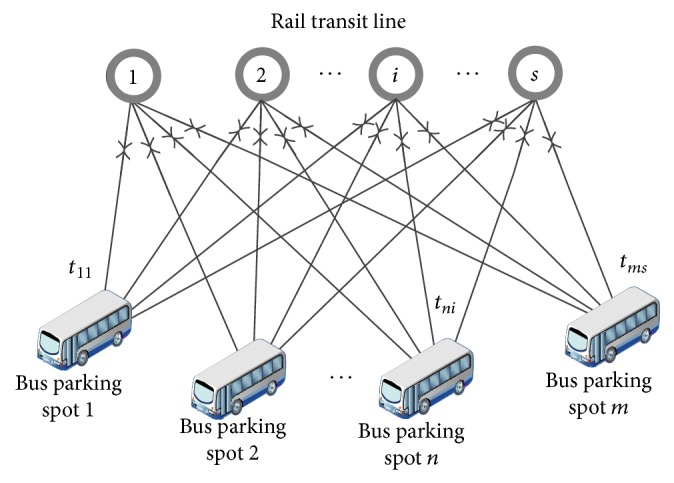
Topological structure when destinations are surrounding bus parking spots.

When the evacuation destinations are surrounding bus parking spots, the task of the dispatched buses is to evacuate stranded passengers to these bus parking spots. The topological structure of this problem is shown in Figure 2. According to the figure, the evacuation process can be described as follows. Firstly, bus parking spot *n* dispatches buses to rail transit station *i* and then passengers get on. Secondly, buses return to their original bus parking spots and passengers get off. Then, buses run circularly between bus parking spot *n* and rail transit station *i*. Finally, the dispatched buses complete the evacuation task and return back to their parking spots. This method is suitable for those rail transit lines with long distances between stations and high operating mileages. However, the passengers will need to transfer to other traffic modes after they have been evacuated to the surrounding bus parking spots.

## 3. Model Development

In order to minimize the effect of the emergency, the evacuation process should be completed as soon as possible. Moreover, due to capacity constraints and cost estimates, the number of dispatched buses should be as few as possible under the premise of satisfying demand. The optimization of the dynamic coscheduling of buses focuses on properly dispatching the reserved vehicles to the bus parking spots.

### 3.1. Model Assumption

The models in this paper are based on the following hypotheses.

(1) The journey time from station *i* to station *k* is equal to the sum of the journey times from station *i* to *j* and from station *j* to *k*:

(1) $$\begin{array}{c} d_{ij}+d_{jk}=d_{ik}\;\left(i<j<k\right). \end{array}$$

(2) The total transportation capability of the bus parking spots can satisfy the evacuation demand.

(3) The buses dispatched from the parking spots have the same properties, and their capacity and speed are fixed values.

### 3.2. Model Building

#### 3.2.1. When the Evacuation Destinations Are Rail Transit Stations

In this case, the dynamic coscheduling model of a line emergency can be described as follows.

*Objective Function.* Minimize the total evacuation time. What makes this study unique is that, as well as the evacuation time, the total number of dispatched buses is also taken into consideration in the optimization objective:

(2) min⁡T=∑n=1m∑i=1ntni+dis+tnsxni +tni+d1i+tn1yni,

where *T* is the total evacuation time, *t*_*ni*_ denotes the minimum journey time from bus parking spot *n* to station *i*, *d*_*ij*_ denotes the minimum journey time from station *i* to station *j*, and *x*_*ni*_ is the number of buses dispatched from bus parking spot *n* to station *i* in the up direction and *y*_*ni*_ is that in the down direction.

*Constraints.* Constraint (3) ensures that the transportation capability of the buses dispatched to each station is more than the evacuation demand of the station, where *C* is the designed seating capacity of the buses and *φ* is the load factor, *A*_*i*_^*on*⁡^ and *A*_*i*_^*off*⁡^, respectively, represent the number of passengers getting on and off the buses in the up direction, and *B*_*i*_^*on*⁡^ and *B*_*i*_^*off*⁡^ are those in the down direction:

(3) C∗ϕ∗∑n=1m∑i=1kxni≥∑i=1kAion⁡−Aioff⁡k=1,2,3,…,s−1,C∗ϕ∗∑n=1m∑i=skyni≥∑i=skBion⁡−Bioff⁡k=s,s−1,s−2,…,2.

Constraint (4) ensures that the number of buses dispatched from each parking spot is less than the number of useable buses in the parking spot, where *N*_*i*_ is the total number of buses in parking spot *n*:

(4) $$\begin{array}{c} \overset{s}{\underset{i=1}{\sum }}\left(x_{ni}+y_{ni}\right)\leq N_{n}\;\left(n=1,2,3,\ldots ,m\right). \end{array}$$

Constraint (5) ensures that the number of buses dispatched from parking spot *n* to station *i* is a positive integer:

(5) $$\begin{array}{c} x_{ni},y_{ni}\geq 0\;\left(n=1,2,\ldots ,m;\;i=1,2,\ldots ,s\right),\\ x_{ni},y_{ni}\in Z\;\left(n=1,2,\ldots ,m;\;i=1,2,\ldots ,s\right). \end{array}$$

#### 3.2.2. When Evacuation Destination Is Bus Parking Spot

When the evacuation destinations are the surrounding bus parking spots, the dynamic coscheduling model for the line emergency can be described as follows.

*Objective Function.* Similarly to the last model, the objective of this model is to minimize the total evacuation time:

(6) $$\begin{array}{c} \min T=\overset{m}{\underset{n=1}{\sum }}\overset{s}{\underset{i=1}{\sum }}\left(k_{ni}+1\right)\left(1+\alpha_{ni}\right)t_{ni}x_{ni}, \end{array}$$

where *k*_*ni*_ denotes the cyclic times of buses being dispatched from parking spot *n* to station *i*, *t*_*ni*_ denotes the minimum journey time from bus parking spot *n* to station *i*, *x*_*ni*_ is the number of buses dispatched from bus parking spot *n* to station *i*, and *α*_*ni*_ means the journey time from bus parking spot *n* to station *i* is *α*_*ni*_ times as that from station *i* to bus parking spot *n*.

*Constraints.* Constraint (7) ensures that the transportation capability of the buses dispatched to each station is more than the evacuation demand of the station, while the capacity is equal to the product of the cyclic times and the number of cyclic buses, where *P*_*i*_ is the number of passengers needing to be evacuated from station *i*:

(7) $$\begin{array}{c} \overset{m}{\underset{n=1}{\sum }}\left(k_{ni}+1\right)x_{ni}\geq \frac{P_{i}}{C*\phi }\;\left(i=1,2,3,\ldots ,s\right). \end{array}$$

Constraint (8) ensures that the cyclic times of the buses are less than the upper limit on cyclic times, where *K* is the upper limit, usually set by the dispatchers:

(8) $$\begin{array}{c} k_{ni}\leq K\;\left(n=1,2,\ldots ,m;\;i=1,2,\ldots ,s\right). \end{array}$$

Constraint (9) ensures that the number of buses dispatched from parking spot *n* to station *i* is a positive integer:

(9) $$\begin{array}{c} x_{ni}\geq 0\;\left(n=1,2,\ldots ,m;\;i=1,2,\ldots ,s\right),\\ x_{ni}\in Z\;\left(n=1,2,\ldots ,m;\;i=1,2,\ldots ,s\right). \end{array}$$

### 3.3. Model Solution

When the evacuation destinations are rail transit stations, the dynamic coscheduling model is an ILP problem, which can be solved directly using the software LINGO.

**Figure 3:**
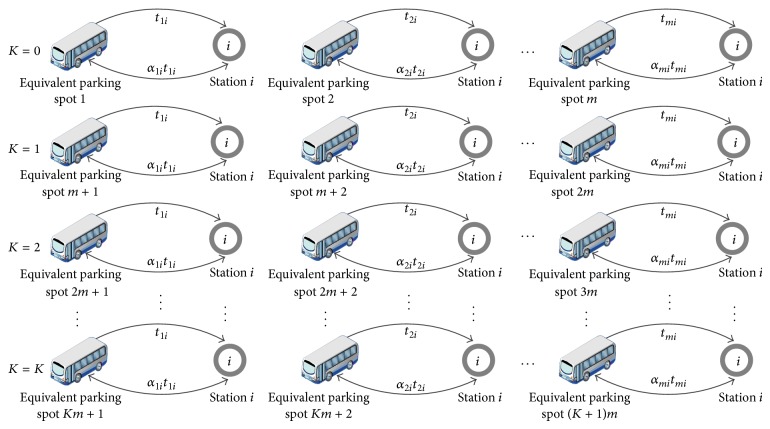
Number of equivalent bus parking spots with different values of *K*.

When the evacuation destinations are the surrounding bus parking spots, the cycle times of buses running between the bus parking spots and the rail transit stations *k*_*ni*_ are not constant in the model. Therefore, the conventional method of integer programming cannot be used to solve this model. To make the model solvable, a concept named the equivalent parking spot is proposed in this paper, with reference to a prior, related study [16]. With the equivalent parking spot, the model can be translated into an IPL problem and the topological structure of the coscheduling of the line emergency is then as shown in Figure 3.

All buses dispatched from the equivalent parking spots are stipulated to evacuate passengers only once, with no buses cycling. In this case, the conversion process of the model can be analyzed as follows.

When the upper limit of the cyclic times is zero, there is only one type of buses, running only once. Therefore, all buses can be regarded as dispatched from equivalent parking spots, the number of which is *m*. When the value of *K* is one, there are two types of buses, one running for once and the other for twice. However, buses dispatched from equivalent parking spots can run only for once. Therefore, each bus running for twice can be regarded as dispatched from two different equivalent parking spots. In other words, each real parking spot should be replaced by two equivalent parking spots. For example, if the number of dispatched buses running between parking spot *n* and station *i* twice is *X*_*ni*_, this is equivalent to the situation where the numbers of buses dispatched from equivalent parking spot *n* and *m* + *i* are both *X*_*ni*_. When the value of *K* increases by one, the type number of running bus will increase by *m*. Therefore, the number of equivalent parking spots will also increase by *m*. Equivalent parking spots are just assumptions and do not exist in reality. Therefore, the minimum journey times from the equivalent bus parking spots to the rail transit station need to be defined. According to Figure 3, the minimum journey times for the equivalent bus parking spots are equal to those for the parking spots from which they are generated. In the previous example, the minimum journey times of equivalent bus parking spots *n* and *m* + *i* are equal to that of parking spot *n*. Regarding the capacities of equivalent bus parking spots, they are the same as the capacities of the original parking spots.

Based on the analysis above, the original model can be transformed into the following model.

*Objective Function.* Minimize the total evacuation time. In this model, the number of equivalent bus parking spots is (*K* + 1)∗*m* and each of the dispatched buses evacuates passengers just once:

(10) $$\begin{array}{c} \min T=\overset{(K+1)m}{\underset{n=1}{\sum }}\overset{s}{\underset{i=1}{\sum }}\left(1+\alpha_{ni}\right)t_{ni}x_{ni}. \end{array}$$

*Constraints.* Constraint (11) ensures that the number of buses dispatched from the equivalent bus parking spots is more than the number of buses needed:

(11) $$\begin{array}{c} \overset{(K+1)m}{\underset{n=1}{\sum }}x_{ni}\geq \frac{P_{i}}{C*\phi }\;\left(i=1,2,3,\ldots ,s\right). \end{array}$$

Constraint (12) ensures that the number of buses dispatched from each equivalent bus parking spot is less than its capacity:

(12) $$\begin{array}{c} \overset{s}{\underset{i=1}{\sum }}x_{ni}\leq N_{n}\;\left(n=1,2,3,\ldots ,\left(K+1\right)m\right). \end{array}$$

Constraint (13) ensures that the number of buses dispatched from the equivalent parking spots to the stations is a positive integer:

(13) $$\begin{array}{c} x_{ni}\geq 0\;\left(n=1,2,\ldots ,\left(K+1\right)m;\;i=1,2,\ldots ,s\right),\\ x_{ni}\in Z\;\left(n=1,2,\ldots ,\left(K+1\right)m;\;i=1,2,\ldots ,s\right). \end{array}$$

After the transformation, the dynamic coscheduling of buses optimization model is a pure ILP problem in operational research, which can be solved using either LINGO or MATLAB software.

## 4. Numerical Analysis

### 4.1. Basic Data

When an unexpected event (e.g., widespread power outages) occurs in a rail transit system, the whole rail transit line is forced to stop operating, leading to a large number of stranded passengers at stations along the line. To ensure passengers' safety, the dynamic coscheduling scheme for buses should be implemented.

In our example, there are four surrounding bus parking spots, each having a different number of available buses; namely, *N*_1_ = 50; *N*_2_ = 30; *N*_3_ = 40; *N*_4_ = 50. Along the rail transit line, there are 12 stations, which are numbered sequentially from 1 to 12. The direction from station 1 to station 12 is denoted as the up direction and that from station 12 to station 1 as the down direction. The numbers of evacuees getting on or off at each station are listed in Table 1.

The minimum journey time *t*_*ni*_ from bus parking spot *n* to station *i* and the time *d*_*ij*_ between station *i* and station *j* are listed in Tables 2 and 3, respectively.

The designed seating capacity of the dispatched buses, *C*, is 100 passengers per bus, and the load factor *φ* is 1.2.

### 4.2. Results of the Dynamic Coscheduling Scheme for Buses

#### 4.2.1. When the Evacuation Destinations Are Rail Transit Stations

Based on the assumed data, the optimal model result is obtained using the LINGO software. The results can be described as follows.

In the up direction, *X*_41_ = 15; *X*_42_ = 10; *X*_43_ = 3. In the down direction, *Y*_110_ = 1; *Y*_410_ = 2; *Y*_411_ = 8; *Y*_412_ = 12. All other variables are zero. The total evacuation time is 4919 minutes.

The results reveal that, in the up direction, bus parking spot 4, respectively, dispatches fifteen, ten, and three buses to rail transit stations 1, 2, and 3. In the down direction, parking spot 1 dispatches only one bus to station 10, while parking spot 4 dispatches two, eight, and twelve buses to stations 10, 11, and 12, respectively. The total number of buses dispatched is 51 and the average evacuation time is about 95 minutes.

#### 4.2.2. When the Evacuation Destinations Are the Surrounding Bus Parking Spots

When the upper limit of the bus cycle times *K* is zero, there is no feasible solution for the optimal model. The results suggest that if no bus runs between the bus parking spots and the rail transit station circularly, all passengers cannot be evacuated.

When the upper limit *K* increases to one, the results are as listed in Table 4.

The results reveal that equivalent bus parking spot 1, respectively, dispatches twelve, sixteen, and eighteen buses to rail transit stations 4, 5, and 6; equivalent parking spot 2, respectively, dispatches five, twelve, and thirteen buses to rail transit stations 1, 2, and 3; equivalent parking spot 4, respectively, dispatches fourteen, twelve, twelve, and twelve buses to rail transit stations 9, 10, 11, and 12; equivalent parking spot 6 dispatches ten buses to rail transit station 1; parking spot 8 dispatches fifteen buses, twenty-one buses, and one bus to rail transit stations 7, 8, and 9. The total evacuation time is 7610 minutes and the total number of dispatched buses is 126. Therefore, the average evacuation time is about 60 minutes.

The result can be explained as follows. Firstly, all the buses are dispatched from equivalent bus parking spots 1 to 4. Secondly, when there are ten buses available in equivalent bus parking spot 6, which also means they are available in spot 2, these ten buses will be dispatched to station 1; similarly, when buses are available in equivalent bus parking spot 8, which also means this number is available in spot 4, the optimized number of buses will be dispatched to stations 7, 8, and 9.

When the upper limit *K* is more than one, the results will remain unchanged. These results suggest that, when the dispatched buses run twice, the transportation capacity is more than sufficient to evacuate all the passengers.

### 4.3. Sensitivity Analysis

Sensitivity analysis is a method of measuring how the uncertainty in the output of a mathematical model can be apportioned to different sources of uncertainty in its inputs [20]. In this paper, sensitivity analysis is conducted to identify the two main influencing factors that have the greatest impact on the dynamic coscheduling scheme for buses. It is found that the designed seating capacity of the dispatched buses and the volume of passengers in the rail transit stations are the two vital factors. Therefore, in the sensitivity analysis, the values of these two parameters are changed with the aim of discovering how much the total evacuation time changes as a result.

**Figure 4:**
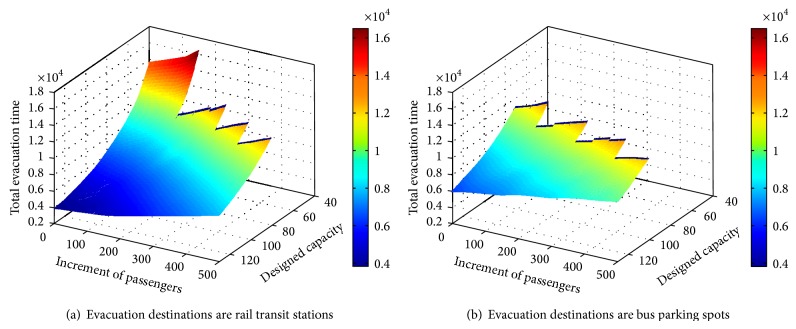
Results of sensitivity analysis.

In the sensitivity analysis, the designed capacity of the buses is increased from 40 passengers per bus to 130, in increments of 10. Similarly, the volume of passengers in each rail transit station is increased in increments of 50 to an upper limit of 500. In each combination of designed conditions, the total evacuation time is calculated. The sensitivity analysis results are shown in Figure 4.

Figure 4 illustrates the relationship between the total evacuation time, the designed capacity of the buses, and the volume of passengers for the two different types of evacuation destination. For both evacuation destinations, the results indicate that the designed capacity of the buses and the volume of passengers have opposite influences on the total evacuation time. With an increase in capacity, the total evacuation time drops quickly, which means that dispatching high-capacity buses will reduce the total evacuation time. However, the downward trend becomes slower as the capacity increases. Inversely, the total evacuation time increases with each increment in the number of passengers and the greater the increment is, the greater the growth rate is. To control the total evacuation time, the dispatching of high-capacity buses should be adopted.

With changes in the parameters, the models may become unsolvable. According to Figure 4, the feasibility of the solution can be described as follows. For rail transit stations, the situation where the model has no solution may happen when the capacity is smaller than 60. When the capacity is 40, no feasible solution can be reached if the increment in the number of passengers is more than 200. If the capacity is increased to 50, the model can be solved up to an increment of 350 passengers. When the capacity increases to 60, unless the number of passengers is increased by 500, the model is solvable. When the capacity is more than 60, there are no unsolvable situations. For surrounding bus parking spots, when the capacity is smaller than 60, even if the increment in the number of passengers is zero, no feasible solution can be obtained. This indicates that, when the capacity of each bus is 40 or 50, the entire capacity of the buses cannot satisfy the demand. When the capacity is 60, there are solutions until the increment in the number of passengers reaches 200. When the capacity increases to 70, the model can be solved up to an increment of 300. When the increment is 500, the entire capacity will not be enough if the capacity of each bus is 80. When the capacity is 90, the model can always be solved no matter how high the increment in the number of passengers is.

Comparing the two graphs, it can be seen that there are more unsolvable situations when the destinations are surrounding parking spots. Furthermore, the total evacuation time of the first model (when the destinations are the surrounding bus parking spots) is much greater than that in the first model (rail transit stations as destinations). This indicates that, under the same given conditions, the bus coscheduling scheme with rail transit stations as destinations performs much better than that with surrounding bus parking spots as destinations because the stability and consequences are much better.

Based on the above analysis, some organization methods and strategies can be proposed to further optimize the bus coscheduling scheme.

(1) Control the quantity of stranded passengers: release information about the emergency that is occurring in the rail transit system in a timely manner to prevent the arrival of new passengers.

(2) Improve the capacity of each bus: dispatch double-decker buses or high-capacity buses.

## 5. Conclusion

URT is one of the most important urban commuter transport modes and always has a high passenger density. Recently, emergencies have occurred frequently on such systems, greatly affecting passenger safety and causing severe traffic delays. Because of the high density, once an emergency occurs, the consequences can be quite serious. However, few researchers have paid attention to the emergency evacuation, not only out of the stations but also to their destinations. Therefore, there is an urgent need to study how passengers should be evacuated and enabled to complete their journeys under emergency conditions.

In this paper, a method of dynamic coscheduling for buses is applied to achieve such as evacuation. Models are built to provide the methodology for designing a bus dynamic coscheduling scheme when the evacuation destinations are, respectively, other rail transit stations and surrounding bus parking spots. Moreover, when the destinations are surrounding bus parking spots, the model is nonlinear. To solve this problem, a new concept of the equivalent parking spot is proposed to transform the nonlinear model into an ILP problem. A case study is conducted to verify the feasibility of the models. The results prove that the model is feasible. The optimized solution makes sense and is consistent with real life.

Finally, the study conducts a sensitivity analysis of two main factors in order to analyze their effects on the total evacuation time. The results reveal that increasing the designed capacity of the dispatched buses reduces the total evacuation time while increasing the number of passengers will increase the total evacuation time. Furthermore, with some changes in the parameters, the models may become unsolvable. Based on the results of the sensitivity analysis, some key strategies are proposed to optimize the bus coscheduling scheme. In summary, this paper may help optimize bus coscheduling and complete the evacuation task with fewer buses and in less time.

## Figures and Tables

**Table 1:** The numbers of evacuated passengers getting on or off.

Stations	1	2	3	4	5	6	7	8	9	10	11	12
*A*_*j*_^*on*⁡^	1800	1200	1000	800	700	1300	800	1200	600	400	200	0
*A*_*j*_^*off*⁡^	0	100	600	900	800	1400	1200	1300	1200	900	1000	600
*B*_*j*_^*on*⁡^	0	200	500	600	1200	800	1000	1300	1200	1000	1200	1400
*B*_*j*_^*off*⁡^	500	800	1000	1000	1300	900	1200	1500	1300	700	200	0

**Table 2:** Minimum journey time *t*_*ni*_ between bus parking spots and stations (min).

Parking spot *n*	Station *i*											
	1	2	3	4	5	6	7	8	9	10	11	12
1	30	27	25	23	20	23	25	30	36	42	50	60
2	28	25	20	24	27	33	38	42	47	50	57	63
3	83	77	65	60	51	46	32	26	21	24	28	32
4	42	36	34	31	29	26	24	21	18	15	21	26

**Table 3:** Minimum journey time *d*_*ij*_ between stations (min).

Station *j*	Station *i*											
	1	2	3	4	5	6	7	8	9	10	11	12
1	0	3	6	9	12	15	18	21	24	27	30	33
2	3	0	3	6	9	12	15	18	21	24	27	30
3	6	3	0	3	6	9	12	15	18	21	24	27
4	9	6	3	0	3	6	9	12	15	18	21	24
5	12	9	6	3	0	3	6	9	12	15	18	21
6	15	12	9	6	3	0	3	6	9	12	15	18
7	18	15	12	9	6	3	0	3	6	9	12	15
8	21	18	15	12	9	6	3	0	3	6	9	12
9	24	21	18	15	12	9	6	3	0	3	6	9
10	27	24	21	18	15	12	9	6	3	0	3	6
11	30	27	24	21	18	15	12	9	6	3	0	3
12	33	30	27	24	21	18	15	12	9	6	3	0

**Table 4:** Results for number of dispatched buses.

Variable	*x*_14_	*x*_15_	*x*_16_	*x*_21_	*x*_22_	*x*_23_	*x*_49_	*x*_410_	*x*_411_	*x*_412_	*x*_61_	*x*_87_	*x*_88_	*x*_89_
Value	12	16	18	5	12	13	14	12	12	12	10	15	21	1
